# Cubature Kalman Hybrid Consensus Filter for Collaborative Localization of Unmanned Surface Vehicle Cluster with Random Measurement Delay

**DOI:** 10.3390/s24186042

**Published:** 2024-09-18

**Authors:** Weicheng Liu, Jichao Yang, Tongbo Xu, Xiaolei Ma, Shengli Wang

**Affiliations:** College of Ocean Science and Engineering, Shandong University of Science and Technology, Qingdao 266590, China; 15266287097@163.com (W.L.); 201901060923@sdust.edu.cn (T.X.); xiaolei_ma_2022@163.com (X.M.); shlwang@sdust.edu.cn (S.W.)

**Keywords:** cubature Kalman filter (CKF), cubature Kalman hybrid consensus filter (CKHCF), collaborative localization, random measurement delays

## Abstract

This paper addresses the collaborative localization problem for unmanned surface vehicle (USV) clusters with random measurement delays. We propose a Cubature Kalman Hybrid Consensus Filter (CKHCF) based on the cubature Kalman filter (CKF) for widely distributed USV clusters lacking global communication capabilities. In this approach, each USV exchanges two pairs of information with all its neighbors and recalculates the received localization data based on distance and relative angle measurements. The recalculated information is then fused with the locally filtered data and updated to obtain localization information based on global measurements. To mitigate the impact of random measurement delays, we employ one-step prediction to compensate for delayed measurements. We present the derivation of the CKHCF algorithm and prove its consistency and boundedness using mathematical induction. Finally, we validate the effectiveness of the proposed algorithm through simulation experiments.

## 1. Introduction

Unmanned surface vehicles (USVs) have gained prominence in ocean exploration, environmental monitoring, and maritime patrol due to their cost-effectiveness, maneuverability, and intelligent capabilities [1,2,3]. However, individual USVs have limited adaptive capacity and struggle to respond effectively to unforeseen events. Consequently, USV clusters are often employed for the collaborative execution of marine tasks in complex and dynamic oceanic environments [4,5]. Collaborative localization technology is crucial for USV clusters, directly impacting navigation accuracy, formation control, and overall performance. Recent years have seen increased attention paid to USV cluster collaborative localization technology [6,7].

Previous research has explored various aspects of USV localization and collaboration. For instance, ref. [8] developed an algorithm for USVs with self-localization capabilities to assist in positioning other USVs lacking this ability. The authors of ref. [9] provided a comprehensive overview of USV collaborative techniques, including formation control and collision avoidance. The authors of ref. [10] proposed a localization method for USV clusters in collaborative formation, although it required maintaining a relatively stable formation. The authors of ref. [11] implemented a sequential fusion filter based on an unscented Kalman filter (UKF) for the real-time localization of individual USVs. The authors of ref. [12] introduced an information fusion algorithm using adaptive fuzzy control to enhance multi-USV system localization accuracy. The authors of ref. [13] presented a networked collaborative dynamic localization control scheme for multiple operating points, ensuring error index convergence within bounded regions. The authors of ref. [14] proposed an integrated positioning and communication-assisted multi-USV network. However, most existing multi-USV collaborative localization methods rely on relatively fixed USV formations, with limited research on collaborative localization for distributed USV clusters without fixed formations.

This paper, therefore, aims to improve localization accuracy and consistency in distributed USV clusters through inter-USV information exchange. We develop a consensus algorithm to integrate localization information from each USV within the cluster.

For distributed network state estimation, three consensus-based information fusion structures exist: Consensus on Estimates (CE), Consensus on Measurement (CM), and Consensus on Information (CI) [15]. CE performs consensus averaging on the state estimates of network nodes at different time points but does not transmit covariance matrix information, hindering state estimation accuracy improvement [16,17,18,19]. CM achieves consensus between local measurements and innovation covariance matrices by fusing information contribution vectors and associated information matrices. However, CM cannot guarantee stability with insufficient consensus steps [20,21]. CI achieves stability for arbitrary consensus steps by fusing information vectors and matrices but ignores correlations between local estimates, potentially reducing estimation accuracy [21,22,23].

To address the limitations of existing consensus fusion methods, ref. [21] introduced the Hybrid Consensus on Measurement and Consensus on Information (HCMCI) fusion method, which combines CM and CI to overcome their respective drawbacks while preserving their advantages. The HCMCI fusion algorithm has since been further developed. The authors of ref. [24] applied HCMCI to target tracking in camera networks. The authors of ref. [25] proposed a hybrid distributed algorithm for consensus tracking in small-scale unmanned aerial vehicle networks based on HCMCI.

In practical engineering applications, most systems are inherently nonlinear, necessitating numerical implementations for nonlinear systems [26,27]. These implementations generally fall into two categories: the direct approximation of nonlinear functions and approximation using probability density functions. The classical Extended Kalman Filter (EKF), proposed in [28], directly expands nonlinear systems using Taylor series. Building on this, ref. [21] introduced the Extended Kalman Hybrid Consensus Filter (EKHCF) for nonlinear systems. The authors of ref. [29] presented the UKF, which reduces errors associated with higher-order Taylor terms, offering improved filtering accuracy compared to the EKF. The authors of ref. [30] proposed the Particle Filter (PF) based on Monte Carlo methods, capable of handling nonlinear models in multidimensional state spaces, albeit with drawbacks such as particle degradation and high computational complexity. The authors of ref. [31] introduced the cubature Kalman filter (CKF) based on the third-degree spherical–radial cubature rule, offering moderate computational requirements and superior filtering accuracy compared to the UKF.

The CKF is widely used in complex nonlinear systems. The authors of ref. [32] propose an indirect fuzzy robust CKF based on a multi-input multi-output fuzzy reasoning system to address the issue of measurement uncertainty in nonlinear systems. To address the issue of inaccurate motion noise statistics caused by dynamic interference in integrated navigation systems, a closed-loop feedback covariance control method based on the CKF is proposed in [33] to solve the aforementioned problem. The authors of ref. [34] propose a CKF-based INS/CNS integrated nonlinear filtering method, which effectively mitigates the adverse effects of non-additive noise and dynamic model uncertainty on inertial measurements. The authors of ref. [35] adopt the principle of multi-model interaction to design a CKF algorithm that combines adaptability and robustness, aiming to solve the tightly coupled integration problem of GNSS/INS. The authors of ref. [36] propose a robust CKF with a scaling factor to mitigate the impact of GNSS observation uncertainty on INS/GNSS integrated navigation systems.

In light of these developments, we propose a Cubature Kalman Hybrid Consensus Filter (CKHCF) based on a CKF and HCMCI to address the collaborative localization problem for widely distributed USV clusters. In this approach, each USV within the cluster initially performs local filtering, then exchanges two sets of information pairs with all neighboring USVs. Corresponding fusion methods are applied to these information pairs, and the position state based on global measurements is calculated through the fused information. Our key contributions are as follows:

(1) The design of the CKHCF algorithm based on the CKF and HCMCI for distributed USV cluster collaborative localization.
(2) The development of a measurement compensation technique to mitigate the impact of random measurement delays.
(3) A proof of the algorithm’s consistency and boundedness for any given time and number of consensus steps using mathematical induction.
(4) A demonstration of the algorithm’s effectiveness through comprehensive simulated experiments.

The remainder of this paper is structured as follows: Section 2 presents the problem formulation. Section 3 details the design and derivation of the CKHCF algorithm for distributed USV cluster collaborative localization. Section 4 provides a proof of the proposed algorithm’s consistency and boundedness using mathematical induction. Section 5 describes a series of simulation experiments and analyzes the results to validate the algorithm’s effectiveness. Section 6 concludes this paper.

Notation: The symbols “−1” and “*T*” denote the matrix inverse and transpose operations, respectively, ‖·‖ denotes the Euclidean norm, and (·) is equal to its previous neighbor. E[·] is the expectation of a random variable, and $diag\{c_{1},c_{2},\cdot \cdot \cdot ,c_{n}\}$ denotes a diagonal matrix with elements $\left\{c_{1},c_{2},\cdot \cdot \cdot ,c_{n}\right\}$.

## 2. Problem Formulation

Since USVs operate solely on the water surface, the positioning coordinate system is simplified to a two-dimensional plane coordinate system.

**Figure 1:**
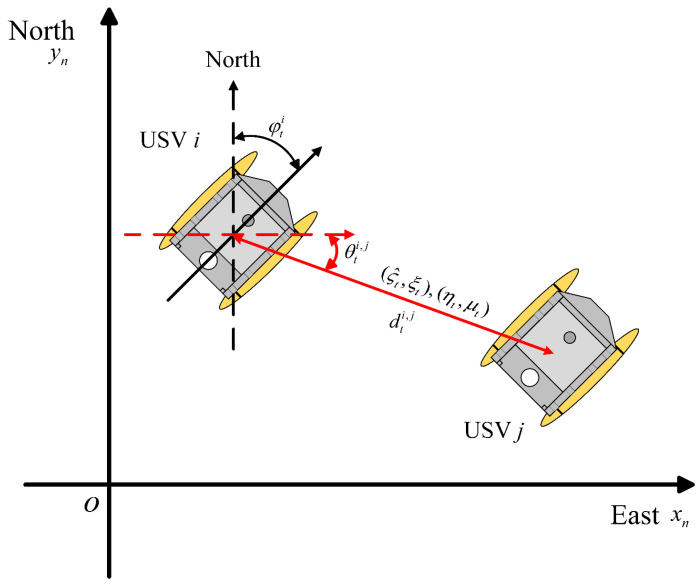
USV two-dimensional plane collaborative localization system.

The local Cartesian coordinate system is chosen as the navigation coordinate system. As depicted in Figure 1, the angle between the carrier coordinate system and the navigation coordinate system represents the heading angle of the USV.

The two-dimensional kinematics model of USV i can be expressed as

(1) $$\left\{ \begin{array}{l} p_{x,t}^{i}=p_{x,t-1}^{i}+v_{t}^{i}\tau_{t}\sin(\varphi_{t}^{i})\\ p_{y,t}^{i}=p_{y.t-1}^{i}+v_{t}^{i}\tau_{t}\cos(\varphi_{t}^{i})\\ \varphi_{t}^{i}=\varphi_{t-1}^{i}+\tau_{t}\omega_{t}^{i},\;i\in N \end{array}\right.$$

where $a_{t}^{i}$ is the forward velocity of USV i at time t, $\omega_{t}^{i}$ is the heading angular velocity of USV i at time t, $\varphi_{t}^{i}$ is the course angle of USV i at time t, $\tau_{t}$ is the dead reckoning epoch, and N is the number of USVs.

This paper aims to enhance the positioning accuracy of each USV by calculating and fusing the position information of different USVs. Consequently, only the location state of USVs will be analyzed [37]. The state vector of the system can be defined as

(2) $$x_{t}^{i}={\left[p_{x,t}^{i}\;p_{y,t}^{i}\right]}^{T}$$

where $p_{x,t}^{i}$ and $p_{y,t}^{i}$ represent the position of USV i at time t in the navigation coordinate system.

From Equations (1) and (2), the nonlinear dynamic model of the system can be expressed as

(3) $$f^{\prime }(x_{t}^{i})=\left(\begin{array}{c} {\dot{p}}_{x,t}^{i}\\ {\dot{p}}_{y,t}^{i} \end{array}\right)=\left(\begin{array}{c} v_{t}^{i}\sin(\varphi_{t}^{i})\\ v_{t}^{i}\cos(\varphi_{t}^{i}) \end{array}\right)$$

From Equation (3), the state equation of the USV cooperative localization system can be expressed as

(4) $$x_{t}^{i}=f(x_{t-1}^{i})+w_{t}^{i}$$

where $x_{t}^{i}$ is the state vector, and $w_{t}^{i}$ is the system noise.

The measurement information of each USV in the collaborative localization system includes its own positional information, as well as the distance and relative angle information between it and other USVs within the communication range.

The measurement information of the USV cluster collaborative localization system with measurement delay can be expressed as

(5) $$\begin{array}{ll} z_{t}^{i} & =\left(\begin{array}{c} p_{x,t}^{i}\\ p_{y,t}^{i}\\ \sqrt{{\left(p_{x,t}^{j}-p_{x,t}^{i}\right)}^{2}+{\left(p_{y,t}^{j}-p_{y,t}^{i}\right)}^{2}}\\ \arctan\left(\frac{p_{y,t}^{j}-p_{y,t}^{i}}{p_{x,t}^{j}-p_{x,t}^{i}}\right) \end{array}\right)+v_{t}^{i}\\ & =\left(\begin{array}{c} p_{x,t}^{i}\\ p_{y,t}^{i}\\ d_{t}^{i,j}\\ \theta_{t}^{i,j} \end{array}\right)+v_{t}^{i}=h(x_{t}^{i})+v_{t}^{i},\;j\in N,j\neq i \end{array}$$

(6) $$y_{t}^{i}=(1-\gamma_{t}^{i})z_{t}^{i}+\gamma_{t}^{i}{\hat{z}}_{t|t-1}^{i},\;t>1;\;y_{t}^{i}=z_{t}^{i}$$

where $z_{t}^{i}$ is the ideal measurement, $y_{t}^{i}$ is the actual measurement, $v_{t}^{i}$ is the measurement noise, $\gamma_{t}^{i}$ is the measured condition, and $d_{t}^{i,j}$ and $\theta_{t}^{i,j}$ represent the distance and relative angle between USV i and USV j.

Different from the measurement equation proposed in [8], we also included measurements of the relative angle between two USVs to ensure the observability of the system when their motion directions are the same during consecutive observation periods.

**Assumption 1.**  *$w_{t}^{i}$ and $v_{t}^{i}$ are Gaussian white noise with zero mean and covariances $Q_{t}^{i}$ and $R_{t}^{i}$, respectively. They satisfy the following*

(7) $$E\left[\left(\begin{array}{c} w_{t}^{i}\\ v_{t}^{i} \end{array}\right)\left(\begin{array}{cc} w_{k}^{l,T} & v_{k}^{l,T} \end{array}\right)\right]=\left[\begin{array}{cc} Q_{t}^{i} & 0\\ 0 & R_{t}^{i} \end{array}\right]\delta_{ij}\delta_{tk}$$

*where $\delta_{ij}$ and $\delta_{tk}$ are Kronecker delta functions.*

**Assumption 2.**  *The random sequence $\gamma_{k}^{i}$ consists of independent Bernoulli random variables that take on the values 1 and 0 with*

(8) $$\begin{array}{l} p(\gamma_{t}^{i}=1)=E[\gamma_{t}^{i}]=p_{t}^{i}\\ p(\gamma_{t}^{i}=0)=1-E[\gamma_{t}^{i}]=1-p_{t}^{i}\\ E[{\left(\gamma_{t}^{i}-p_{t}^{i}\right)}^{2}]=(1-p_{t}^{i})p_{t}^{i} \end{array}$$

*where $p_{t}^{i}$ is the probability of the USV i measurement delay at time t.*

**Assumption 3.**  *The initial state vector ${\hat{x}}_{0}^{i}$ is independent of $w_{t}^{i}$, $v_{t}^{i}$, and $\gamma_{t}^{i}$ and satisfies the following equation.*

(9) $$E[x_{0}^{i}]={\hat{x}}_{0}^{i}$$

(10) $$E[(x_{0}^{i}-{\hat{x}}_{0}^{i}){\left(x_{0}^{i}-{\hat{x}}_{0}^{i}\right)}^{T}]=P_{0}^{i}=\zeta_{0}^{i,-1}$$

*where $P_{0}^{i}$ and $\zeta_{0}^{i}$ are the initial error covariance matrix and information matrix.*

The objective of this paper is to design a collaborative localization algorithm for distributed USV clusters with random measurement delays.

## 3. USV Cluster Collaborative Localization Algorithm

In this section, we design a CKHCF collaborative localization algorithm for a large-scale distributed USV cluster with random measurement delays.

In a large-scale distributed USV cluster, each USV within the cluster can only exchange information with its adjacent USVs due to limitations such as distance and the lack of global communication capability.

The communication structure of a cluster network consisting of USVs can be represented by a topology graph G=(V,E) with a USV set V={1,2,⋯,N} and an edge set E⊆V×V. If information can be exchanged between USVs i and j, then they are neighbors, and we define the neighboring USVs of USV i as $N_{i}=\{j|(i,j)\in E\}$. The graph is strongly connected.

**Figure 2:**
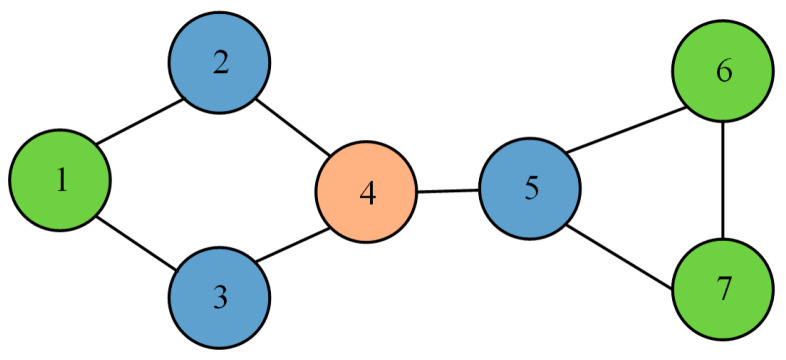
Communication network topology with seven USVs.

Figure 2 shows the communication network topology for the USV cluster with seven USVs. Taking USV 4 as an example, two consensus fusion steps are required to acquire global information. In the first consensus fusion step, the position information from USVs 2, 3, and 5 is obtained. In the second consensus fusion step, the position information containing USVs 1, 6, and 7 is obtained.

In Assumption 1, for the sake of derivation convenience, we assume that the USV cluster collaborative localization system is a noise-independent system, as shown in (7), where system noise is unrelated to measurement noise, the measurement noise of each USV is independent, and all noises are independent of their previous time step. In Assumption 2, we assume that the probability of a measurement delay in a USV cluster is $\gamma_{t}^{i}$. Equation (8) can be obtained from probability statistics. In Assumption 3, we assume that the initial state vector is independent of the system noise, measurement noise, and measured condition in the filtering process, and its error covariance matrix is the initial matrix of error covariance. The following theorems and conclusions are derived based on Assumptions 1–3.

**Theorem 1.**  *For Systems (4), (5), and (6) under the conditions of Assumptions 1–3, we have the first-stage USV cluster collaborative localization algorithm CKHCF with local filter pairs $\left({\hat{\varsigma }}_{t,0}^{i},\zeta_{t,0}^{i}\right)$* *and* $\left({\hat{\eta }}_{t,0}^{i},\mu_{t,0}^{i}\right)$.

**Proof of Theorem 1.**  In the first stage, each USV independently conducts local filtering based on its own measurements. □

(11) $${\hat{\varsigma }}_{t,0}^{i}={\hat{\varsigma }}_{t|t-1,0}^{i}+\zeta_{t|t-1,0}^{i}P_{xy,t|t-1}^{i}{\left(R_{t}^{i}\right)}^{-1}[{\tilde{y}}_{t|t-1}^{i}+{\left(P_{xy,t|t-1}^{i}\right)}^{T}\zeta_{t|t-1,0}^{i}{\hat{x}}_{t|t-1}^{i}]$$

(12) $$\zeta_{t,0}^{i}=\zeta_{t|t-1,0}^{i}+\zeta_{t|t-1,0}^{i}P_{xy,t|t-1}^{i}{\left(R_{t}^{i}\right)}^{-1}{\left(P_{xy,t|t-1}^{i}\right)}^{T}\zeta_{t|t-1,0}^{i}$$

(13) $${\hat{\eta }}_{t,0}^{i}=\zeta_{t|t-1,0}^{i}P_{xy,t|t-1}^{i}{\left(R_{t}^{i}\right)}^{-1}[{\tilde{y}}_{t|t-1}^{i}+{\left(P_{xy,t|t-1}^{i}\right)}^{T}\zeta_{t|t-1,0}^{i}{\hat{x}}_{t|t-1}^{i}]$$

(14) $$\mu_{t,0}^{i}=\zeta_{t|t-1,0}^{i}P_{xy,t|t-1}^{i}{\left(R_{t}^{i}\right)}^{-1}{\left(P_{xy,t|t-1}^{i}\right)}^{T}\zeta_{t|t-1,0}^{i}$$

where $\left({\hat{\varsigma }}_{t,0}^{i},\zeta_{t,0}^{i}\right)$ is an information pair composed of local filter information state vector ${\hat{\varsigma }}_{t,0}^{i}$ and information matrix $\zeta_{t,0}^{i}$, $\left({\hat{\eta }}_{t,0}^{i},\mu_{t,0}^{i}\right)$ is another information pair composed of local filter information contribution state vector ${\hat{\eta }}_{t,0}^{i}$ and its associated information matrix $\mu_{t,0}^{i}$, ${\tilde{y}}_{t|t-1}^{i}$ is the error of predicted measurement, ${\hat{\varsigma }}_{t|t-1,0}^{i}$ and $\zeta_{t|t-1,0}^{i}$ are the predicted information vector and information matrix, and $P_{xy,t|t-1}^{i}$ is the cross-covariance matrix at time t−1.

The predicted measurement error ${\tilde{y}}_{t|t-1}^{i}$ can be expressed as

(15) $${\tilde{y}}_{t|t-1}^{i}=(1-\gamma_{t}^{i})z_{t}^{i}+(\gamma_{t}^{i}-1){\hat{z}}_{t|t-1}^{i}$$

(16) $${\hat{z}}_{t|t-1}^{i}=\int h^{i}(x_{t}^{i})N(x_{t}^{i};{\hat{x}}_{t|t-1}^{i},\zeta_{t|t-1,0}^{i,-1})dx_{t}^{i}$$

where ${\hat{x}}_{t|t-1}^{i}$ is the predicted state, and ${\hat{z}}_{t|t-1}^{i}$ is the predicted ideal measurement.

The predicted state ${\hat{x}}_{t|t-1}^{i}$ can be expressed as

(17) $${\hat{x}}_{t|t-1}^{i}=\int f(x_{t-1}^{i})N(x_{t-1}^{i};{\hat{x}}_{t-1,L}^{i},\zeta_{t-1,L}^{i,-1})dx_{t-1}^{i}$$

where ${\hat{x}}_{t-1,L}^{i}$ and $\zeta_{t-1,L}^{i,-1}$ are the state vector and information matrix based on global measurements at time t−1.

The predicted information vector ${\hat{\varsigma }}_{t|t-1,0}^{i}$ and information matrix $\zeta_{t|t-1,0}^{i}$ can be expressed as

(18) $$\zeta_{t|t-1,0}^{i}={\left(\int f(x_{t-1}^{i}){\left(\cdot \right)}^{T}N(x_{t-1}^{i};{\hat{x}}_{t-1,L}^{i},\zeta_{t-1,L}^{i,-1})dx_{t-1}^{i}-{\hat{x}}_{t|t-1}^{i}{\hat{x}}_{t|t-1}^{i,T}+Q_{t}^{i}\right)}^{-1}$$

(19) $${\hat{\varsigma }}_{t|t-1,0}^{i}=\zeta_{t|t-1,0}^{i}{\hat{x}}_{t|t-1}^{i}$$

The cross-covariance $P_{xy,t|t-1}^{i}$ can be expressed as

(20) $$P_{xy,t|t-1}^{i}=(1-p_{t}^{i})\int x_{t}^{i}{\left(h^{i}(x_{t}^{i})\right)}^{T}N(x_{t}^{i};{\hat{x}}_{t|t-1}^{i},\zeta_{t|t-1,0}^{i,-1})dx_{t}^{i}-(1-p_{t}^{i}){\hat{x}}_{t|t-1}^{i}{\hat{z}}_{t|t-1}^{i,T}$$

The local filter information matrix $\zeta_{t,0}^{i}$ and information vector ${\hat{\varsigma }}_{t,0}^{i}$ are defined as

(21) $$\zeta_{t,0}^{i}={\left(P_{t,0}^{i}\right)}^{-1}$$

(22) $${\hat{\varsigma }}_{t,0}^{i}={\left(P_{t,0}^{i}\right)}^{-1}{\hat{x}}_{t,0}^{i}=\zeta_{t,0}^{i}{\hat{x}}_{t,0}^{i}$$

where $P_{t,0}^{i}$ is the local filter state error covariance matrix of USV i at time t.

From the Kalman Gaussian filter, the local iteration of information vector ${\hat{\varsigma }}_{t,0}^{i}$ and information matrix $\zeta_{t,0}^{i}$ can be calculated as (11).

Then, the local iteration of information state contribution vector $\eta_{t,0}^{i}$ and associated information matrix $\mu_{t,0}^{i}$ are defined as (13).

The predicted measurement can be calculated as

(23) $${\hat{y}}_{t|t-1}^{i}=E[(1-\gamma_{t}^{i})z_{t}^{i}+\gamma_{t}^{i}{\hat{z}}_{t|t-1}^{i}]={\hat{z}}_{t|t-1}^{i}$$

(24) $${\hat{z}}_{t|t-1}^{i}=E[z_{t}^{i}]=\int h^{i}(x_{t}^{i})N(x_{t}^{i};{\hat{x}}_{t|t-1}^{i},\zeta_{t-1,L}^{i,-1})dx_{t}^{i}$$

The predicted measurement error ${\tilde{y}}_{t|t-1}^{i}$ can be calculated as

(25) $$\begin{array}{ll} {\tilde{y}}_{k+1|k}^{i} & =y_{k+1}^{i}-{\hat{y}}_{k+1|k}^{i}\\ & =(1-\gamma_{t}^{i})z_{t}^{i}+\gamma_{t}^{i}{\hat{z}}_{t|t-1}^{i}-{\hat{z}}_{t|t-1}^{i}\\ & =(1-\gamma_{t}^{i})z_{t}^{i}+(\gamma_{t}^{i}-1){\hat{z}}_{t|t-1}^{i} \end{array}$$

The cross-covariance $P_{xy,t|t-1}^{i}$ can be calculated as

(26) $$\begin{array}{ll} P_{t|t-1}^{x^{i}y^{i}} & =E[(x_{t}^{i}-{\hat{x}}_{t|t-1}^{i}){\left(y_{t}^{i}-{\hat{y}}_{t|t-1}^{i}\right)}^{T}]\\ & =E[(x_{t}^{i}-{\hat{x}}_{t|t-1}^{i}){\left((1-\gamma_{t}^{i})z_{t}^{i}+(\gamma_{t}^{i}-1){\hat{z}}_{t|t-1}^{i}\right)}^{T}]\\ & =(1-p_{t}^{i})E[x_{t}^{i}{\left(h^{i}(x_{t}^{i})\right)}^{T}]-(1-p_{t}^{i}){\hat{x}}_{t|t-1}^{i}E[z_{t}^{i,T}]\\ & \;-(1-p_{t}^{i})E[x_{t}^{i}]{\hat{z}}_{t|t-1}^{i,T}+(1-p_{t}^{i}){\hat{x}}_{t|t-1}^{i}{\hat{z}}_{t|t-1}^{i,T}\\ & =(1-p_{t}^{i})\int x_{t}^{i}{\left(h^{i}(x_{t}^{i})\right)}^{T}N(x_{t}^{i};{\hat{x}}_{t|t-1}^{i},\zeta_{t|t-1,0}^{i,-1})dx_{t}^{i}-(1-p_{t}^{i}){\hat{x}}_{t|t-1}^{i}{\hat{z}}_{t|t-1}^{i,T} \end{array}$$

From Equation (21), the information state vector ${\hat{\varsigma }}_{t|t-1}^{i}$ can be calculated as (18), and the predicted information matrix $\zeta_{t|t-1}^{i}$ can be calculated as

(27) $$\begin{array}{ll} \zeta_{t|t-1}^{i} & =P_{t|t-1}^{i,-1}=E^{-1}[(x_{t}^{i}-{\hat{x}}_{t|t-1}^{i}){\left(x_{t}^{i}-{\hat{x}}_{t|t-1}^{i}\right)}^{T}]\\ & ={\left(E[x_{t}^{i}x_{t}^{i,T}]-E[x_{t}^{i}]{\hat{x}}_{t|t-1}^{i,T}-{\hat{x}}_{t|t-1}^{i}E[x_{t}^{i,T}]+{\hat{x}}_{t|t-1}^{i}{\hat{x}}_{t|t-1}^{i,T}\right)}^{-1}\\ & ={\left(\int f(x_{t-1}^{i}){\left(\cdot \right)}^{T}N(x_{t-1}^{i};{\hat{x}}_{t-1,L}^{i},\zeta_{t-1,L}^{i,-1})dx_{k}-{\hat{x}}_{t|t-1}^{i}{\hat{x}}_{t|t-1}^{i,T}+Q_{t}^{i}\right)}^{-1} \end{array}$$

where ${\hat{x}}_{t-1,L}^{i}$ and $\zeta_{t-1,L}^{i,-1}$ will be defined in Theorem 2.

**Theorem 2.**  *For Systems (4), (5), and (6) with assumptions 1–3, the second-stage USV cluster cooperative localization algorithm CKHCF can be expressed as follows. For Systems (4), (5), and (6) under the conditions of Assumptions 1–3, we have the first-stage USV cluster collaborative localization algorithm CKHCF with mean ${\hat{x}}_{t,L}^{i}$ and information covariance matrix* $\zeta_{t,L}^{i}$.

**Proof of Theorem 2.**  In the second stage, all USVs transmit information pairs $\left({\hat{\varsigma }}_{t|t-1,l}^{i},\zeta_{t|t-1,l}^{i}\right)$ and $\left(\eta_{t,l}^{i},\mu_{t,l}^{i}\right)$, i∈N, l=0,1,⋯,L−1 (l denotes the consensus fusion steps, L is the number of consensus fusion steps) to their neighboring USVs and receive two sets of information pairs from all their neighboring USVs. Subsequently, the state vectors within the information pairs are calculated using measured distances and relative angles and then fused with their own filter results. □

As shown in Figure 1, USV i recalculates the information vector of the received information through measurement. The recalculated information vector can be calculated as

(28) $${\hat{\varsigma }}_{t|t-1,l}^{i,j}={\hat{\varsigma }}_{t|t-1,l}^{j}-\zeta_{t|t-1,l}^{j}\left[\begin{array}{c} d_{t}^{i,j}\sin(\theta_{t}^{i,j})\\ d_{t}^{i,j}\cos(\theta_{t}^{i,j}) \end{array}\right]$$

(29) $${\hat{\eta }}_{t,l}^{i,j}=\zeta_{t|t-1,l}^{j}P_{xy,t|t-1}^{j}{\left(R_{t}^{j}\right)}^{-1}P_{xy,t|t-1}^{j,T}\zeta_{t|t-1,l}^{j}\left[\begin{array}{c} d_{t}^{i,j}\sin(\theta_{t}^{i,j})\\ d_{t}^{i,j}\cos(\theta_{t}^{i,j}) \end{array}\right]$$

The recalculated state vectors are substituted into the information pairs received by USV i and denoted as $\left({\hat{\varsigma }}_{t|t-1,l}^{j},\zeta_{t|t-1,l}^{j}\right)$ and $\left({\hat{\eta }}_{t,l}^{j},\mu_{t,l}^{j}\right)$, $j\in N_{i}$.

The fusion of two information pairs can be calculated as

(30) $${\hat{\varsigma }}_{t|t-1,l+1}^{i}=\sum_{j\in N_{i}}G^{i,j}{\hat{\varsigma }}_{t|t-1,l}^{i,j}$$

(31) $$\zeta_{t|t-1,l+1}^{i}=\sum_{j\in N_{i}}G^{i,j}\zeta_{t|t-1,l}^{j}$$

(32) $${\hat{\eta }}_{t,l+1}^{i}=\sum_{j\in N_{i}}G^{i,j}{\hat{\eta }}_{t,l}^{i,j}$$

(33) $$\mu_{t,l+1}^{i}=\sum_{j\in N_{i}}G^{i,j}\mu_{t,l}^{j}$$

where $G^{i,j}$, $j\in N_{i}$ is the fusion weight, and $\sum_{j\in N_{i}}G^{i,j}=1$.

The information vector and information matrix are updated as

(34) $${\hat{\varsigma }}_{t,L}^{i}={\hat{\varsigma }}_{t|t-1,L}^{i}+\lambda_{t}^{i}{\hat{\eta }}_{t,L}^{i}$$

(35) $$\zeta_{t,L}^{i}=\zeta_{t|t-1,L}^{i}+\lambda_{t}^{i}\mu_{t,L}^{i}$$

where $\lambda_{t}^{i}$ is the consensus scalar weight.

Take $\lambda_{t}^{i}=|N|$, which represents the cardinality of the set N. When consensus step L is large enough, the consensus weights are chosen $G^{i,j}\to 1/|N|$ which can provide ${\hat{\eta }}_{t,L}^{i}\to \frac{1}{N}\sum_{t=1}^{N}{\hat{\eta }}_{t}^{i}$ and $\mu_{t,L}^{i}\to \frac{1}{N}\sum_{t=1}^{N}\mu_{t}^{i}$; therefore, the accuracy of this distributed algorithm converges to that of a centralized algorithm.

Then, the state vector after information fusion ${\hat{x}}_{t,L}^{i}$ can be calculated as

(36) $${\hat{x}}_{t,L}^{i}={\left(\zeta_{t,L}^{i}\right)}^{-1}{\hat{\varsigma }}_{t,L}^{i}$$

The numerical implementation of ${\hat{x}}_{t|t-1}^{i}$, ${\hat{\varsigma }}_{t|t-1,0}^{i}$, $\zeta_{t|t-1,0}^{i}$, and $P_{xy,t|t-1}^{i}$ is as follows

1. The covariance is factorized
  (37) $${\left(\zeta_{t-1,L}^{i}\right)}^{-1}=S_{t-1}^{i}{\left(S_{t-1}^{i}\right)}^{T}$$
2. The cubature points are evaluated (k=1,2,⋯,2m)
  (38) $$X_{k,t-1}^{i}={\hat{x}}_{t-1,L}^{i}+S_{t-1}^{i}\gamma_{k}$$
  where the unit cubature point is defined as
  (39) $$u_{k}=\left\{ \begin{array}{ll} \sqrt{m}e_{k}, & k=1,2,\cdots ,m\\ -\sqrt{m}e_{k-m}, & k=m+1,\cdots ,2m \end{array}\right.$$
  where $e_{k}$ is the n-dimensional unit vector with the kth element.
3. The propagated cubature points are evaluated (k=1,2,⋯,2m)
  (40) $$X_{k,t|t-1}^{i}=f(X_{k,t-1}^{i})$$
4. ${\hat{x}}_{t|t-1}^{i}$, ${\hat{\varsigma }}_{t|t-1,0}^{i}$, and $\zeta_{t|t-1,0}^{i}$ are estimated
  (41) $${\hat{x}}_{t|t-1}^{i}=\frac{1}{2m}\sum_{k=1}^{2m}X_{k,t|t-1}^{i}$$
  (42) $$\zeta_{t|t-1,0}^{i}={\left[\frac{1}{2m}\sum_{k=1}^{2m}X_{k,t|t-1}^{i}{\left(\cdot \right)}^{T}-{\hat{x}}_{t|t-1}^{i}{\left(\cdot \right)}^{T}+Q_{t}^{i}\right]}^{-1}$$
  (43) $${\hat{\varsigma }}_{t|t-1,0}^{i}=\zeta_{t|t-1,0}^{i}{\hat{x}}_{t|t-1}^{i}$$
5. The prediction covariance is factorized
  (44) $${\left(\zeta_{t|t-1,0}^{i}\right)}^{-1}=S_{t|t-1}^{i}{\left(S_{t|t-1}^{i}\right)}^{T}$$
6. The prediction cubature points are evaluated (k=1,2,⋯,2m)
  (45) $$X_{k,t|t-1}^{i}={\hat{x}}_{t|t-1}^{i}+S_{t|t-1}^{i}u_{k}$$
7. The propagated prediction cubature points are evaluated (k=1,2,⋯,2m)
  (46) $$Y_{k,t|t-1}^{i}=h(X_{k,t|t-1}^{i})$$
8. The prediction measurement is estimated
  (47) $${\hat{y}}_{t|t-1}^{i}=\frac{1}{2m}\sum_{i=1}^{2m}Y_{k,t|t-1}^{i}$$
9. The cross-covariance matrix is estimated
  (48) $$P_{xy,t|t-1}^{i}=\frac{1}{2m}\sum_{k=1}^{2m}X_{k,t|t-1}^{i}{\left(Y_{k,t|t-1}^{i}\right)}^{T}-{\hat{x}}_{t|t-1}^{i}{\left({\hat{y}}_{t|t-1}^{i}\right)}^{T}$$

The collaborative localization algorithm for a USV cluster, based on CKHCF and applicable to a wide range of distributions, can be summarized as follows (Algorithm 1).
**Algorithm 1** CKHCFGiven initial values ${\hat{x}}_{0}^{i}={\left[p_{x,0}^{i}p_{y,0}^{i}\right]}^{T}\;\mathrm{and}\;\zeta_{0}^{i}=P_{0}^{-1}.$Step 1. (First stage) Each USV performs local filtering. Measurements $y_{t-1}^{i},i\in N$ are obtained for each USVs. Calculate ${\hat{x}}_{t|t-1}^{i}$ by Equation (41). Calculate ${\tilde{y}}_{t|t-1}^{i}$ by Equation (15). Calculate $P_{xy,t|t-1}^{i}$ by Equation (48). Calculate the predicted information pair $\left({\hat{\varsigma }}_{t|t-1,0}^{i},\zeta_{t|t-1,0}^{i}\right)$ by Equations (18) and (19). Calculate the information pair $\left(\eta_{t,0}^{i},\mu_{t,0}^{i}\right)$ by Equations (13) and (14).Step 2. (Second stage) Information pairs exchange and fusion. For *l* = 0, 1, …, *L* − 1, implement the following consensus steps in parallel. (1) Each USV transmits information pairs $\left({\hat{\varsigma }}_{t|t-1,l}^{i},\zeta_{t|t-1,l}^{i}\right)\;\mathrm{and}\;(\eta_{t,l}^{i},\mu_{t,l}^{i}),\;i\in N\;\mathrm{to}\;\mathrm{its}\;\mathrm{neighbor}\;\mathrm{USVs}\;j\in N_{i}.$ (2) $\mathrm{Each}\;\mathrm{USV}\;\mathrm{receives}\;\mathrm{information}\;\mathrm{pairs}\;({\hat{\varsigma }}_{t|t-1,l}^{j},\zeta_{t|t-1,l}^{j})\;\mathrm{and}\;({\hat{\eta }}_{t,l}^{j},\mu_{t,l}^{j})\;\mathrm{from}\;\mathrm{all}\;\mathrm{neighbors}\;j\in N_{i}.$ (3) $\mathrm{Calculate}\;\mathrm{the}\;\mathrm{information}\;\mathrm{vector}\;{\hat{\varsigma }}_{t|t-1,l}^{i,j}$ by Equation (28). (4) $\mathrm{Each}\;\mathrm{USV}\;\mathrm{fuses}\;\mathrm{two}\;\mathrm{information}\;\mathrm{pairs}\;({\hat{\varsigma }}_{t|t-1,l+1}^{i},\zeta_{t|t-1,l+1}^{i})\;\mathrm{and}\;(\eta_{t,l+1}^{i},\mu_{t,l+1}^{i})$ by Equations (30)–(33). Information update and state vector settlement. $\mathrm{Update}\;\mathrm{information}\;\mathrm{vector}\;{\hat{\varsigma }}_{t,L}^{i}\;\mathrm{and}\;\mathrm{information}\;\mathrm{matrix}\;\zeta_{t,L}^{i}$ by Equations (34) and (35). $\mathrm{Calculate}\;\mathrm{the}\;\mathrm{fused}\;\mathrm{state}\;\mathrm{vector}\;{\hat{x}}_{t,L}^{i}$ by Equation (36).Step 3. Let *t* = *t* + 1, return to step 1.

**Remark 1.**  *When the USV cluster is distributed within a limited area, each USV has global communication capability. In this case, the USVs in the cluster are all neighbors and can be considered as a special case with a consensus step of 1.*

## 4. Stability Analysis

In this section, we demonstrate the stability of the proposed algorithm from two aspects: consistency and boundedness.

### 4.1. System Linearization Approximation

In order to facilitate analysis, the nonlinear system is linearized.

The method of statistical linear error propagation is first used to construct the state transition matrix $F_{t}^{i}$ and measurement matrix $H_{t}^{i}$. According to [15], $F_{t}^{i}$ and $H_{t}^{i}$ can be calculated as

(49) $$H_{t}^{i}={\left(P_{xy,t|t-1}^{i}\right)}^{T}\zeta_{t|t-1}^{i}$$

(50) $$F_{t-1}^{i}={\left(P_{x_{t-1},x_{t|t-1}}^{i}\right)}^{T}\zeta_{t-1}^{i}$$

where $P_{xy,t|t-1}^{i}$ can be calculated by Equation (48), and $P_{x_{t-1},x_{t|t-1}}^{i}$ can be calculated as

(51) $$P_{x_{t-1},x_{t|t-1}}^{i}=\frac{1}{2m}\sum_{k=1}^{2m}(X_{k,t-1}^{i}-{\hat{x}}_{t-1}^{i}){\left(X_{k,t|t-1}^{i}-{\hat{x}}_{t|t-1}^{i}\right)}^{T}$$

Two unknown instrument diagonal matrices $\alpha_{t-1}^{i}=diag(\alpha_{t-1,1}^{i},\alpha_{t-1,2}^{i},\cdots ,\alpha_{t-1,n}^{i})$ and $\beta_{t}^{i}=diag(\beta_{t,1}^{i},\beta_{t,2}^{i},\cdots ,\beta_{t,n}^{i})$ are defined, which are introduced to compensate for the higher-order terms of the Taylor expansion.

Therefore, Systems (4) and (5) can be approximated by linearization as

(52) $$x_{t}^{i}=\alpha_{t-1}^{i}F_{t-1}^{i}x_{t-1}^{i}+w_{t}^{i}$$

(53) $$y_{t}^{i}=\beta_{t}^{i}H_{t}^{i}x_{t}^{i}+v_{t}^{i}$$

Based on linear systems (52) and (53), the information filter can be rewritten as

(54) $${\hat{\varsigma }}_{t|t-1}^{i}=\zeta_{t|t-1}^{i}(\alpha_{t-1}^{i}F_{t-1}^{i}){\hat{\varsigma }}_{t-1}^{i}$$

(55) $$\zeta_{t|t-1}^{i}={\left[\alpha_{t-1}^{i}F_{t-1}^{i}{\left(\zeta_{t-1}^{i}\right)}^{-1}{\left(\alpha_{t-1}^{i}F_{t-1}^{i}\right)}^{T}+Q_{t-1}^{i}\right]}^{-1}$$

(56) $${\hat{\varsigma }}_{t}^{i}={\hat{\varsigma }}_{t|t-1}^{i}+{\left(\beta_{t}^{i}H_{t}^{i}\right)}^{T}{\left(R_{t}^{i}\right)}^{-1}y_{t}^{i}$$

(57) $$\zeta_{t}^{i}=\zeta_{t|t-1}^{i}+{\left(\beta_{t}^{i}H_{t}^{i}\right)}^{T}{\left(R_{t}^{i}\right)}^{-1}\beta_{t}^{i}H_{t}^{i}$$

### 4.2. Proof of Consensus

**Lemma 1.**  *Consider a random vector x, and let $\hat{x}$ and P be the unbiased estimates and error covariance of x, respectively. When $\hat{x}$ and P satisfy the following equation,*

(58) $$E[(x-\hat{x}){\left(x-\hat{x}\right)}^{T}]\leq P$$

*$\left(\hat{\varsigma },\zeta )=(P^{-1}x,P^{-1}\right)$ can be considered consistent [38].*

**Lemma 2.**  *Define the function C(·) to be monotonic and non-decreasing. Function C(·) is determined by the following formula.*

(59) $$\zeta_{t|t-1}^{i}=C(\zeta_{t-1}^{i})$$

**Theorem 3.**  *When the initial error covariance satisfies*

(60) $$\zeta_{1|0}^{i}\leq E^{-1}[(x_{1}-{\hat{x}}_{1|0}^{i}){\left(x_{1}-{\hat{x}}_{1|0}^{i}\right)}^{T}]$$

*the algorithm CKHCF can maintain consistency in any consensus step at any time.*

(61) $$\zeta_{t}^{i}\leq E^{-1}[(x_{t}^{i}-{\hat{x}}_{t}^{i}){\left(x_{t}^{i}-{\hat{x}}_{t}^{i}\right)}^{T}]$$

**Proof of Theorem 3.**  Assume that at time k, the following equation holds,

(62) $$\zeta_{t|t-1}^{i}\leq E^{-1}[(x_{t}^{i}-{\hat{x}}_{t|t-1}^{i}){\left(x_{t}^{i}-{\hat{x}}_{t|t-1}^{i}\right)}^{T}],i\in N$$

From the choice of weight $\lambda_{t}^{i}$, it can be inferred that $\lambda_{t}^{i}\geq 1$. We can obtain

(63) $$\begin{array}{ll} \zeta_{t,1}^{i} & =\sum_{j\in N_{i}}G^{ij}[\zeta_{t|t-1,0}^{j}+\lambda_{t}^{i}\mu_{t,0}^{i}]\\ & \geq \sum_{j\in N_{i}}G^{ij}[\zeta_{t|t-1,0}^{j}+\mu_{t,0}^{i}] \end{array}$$

Using the initialization algorithm, from Equation (62), we can obtain

(64) $$\begin{array}{ll} E^{-1}[(x_{t}^{i}-{\hat{x}}_{t,0}^{i}){\left(x_{t}^{i}-{\hat{x}}_{t,0}^{i}\right)}^{T}] & \geq \zeta_{t|t-1,0}^{i}+\lambda_{t}^{i}\mu_{t,0}^{i}\\ & \geq \zeta_{t|t-1,0}^{i}+\mu_{t,0}^{i}=\zeta_{t,0}^{i} \end{array}$$

Since the covariance intersection fusion rule is consistent, the following can be obtained

(65) $$E^{-1}[(x_{t}^{i}-{\hat{x}}_{t,l+1}^{i}){\left(x_{t}^{i}-{\hat{x}}_{t,l+1}^{i}\right)}^{T}]\geq \zeta_{t,l+1}^{i},\;l=0,1,\cdots ,L-1$$

From Equation (65) and Lemma 2, we can obtain

(66) $$\begin{array}{ll} \zeta_{t+1|t,0}^{i} & =C(\zeta_{t,L}^{i})\\ & \leq C(E^{-1}[(x_{t}^{i}-{\hat{x}}_{t,L}^{i}){\left(x_{t}^{i}-{\hat{x}}_{t,L}^{i}\right)}^{T}])\\ & =E^{-1}[(x_{t+1}^{i}-{\hat{x}}_{t+1|t}^{i}){\left(x_{t+1}^{i}-{\hat{x}}_{t+1|t}^{i}\right)}^{T}] \end{array}$$

According to mathematical induction, when the initial error covariance satisfies (60), the following inequalities hold true

(67) $$\zeta_{t,l}^{i}\leq E^{-1}[(x_{t}-x_{t,l}^{i}){\left(x_{t}-x_{t,l}^{i}\right)}^{T}],\;i\in N,l\in N_{i}$$

In conclusion, the Proof of Theorem 3 is completed. □

### 4.3. Proof of Boundedness

**Theorem 4.**  *If the initial error covariance matrix $P_{0}^{i}=\zeta_{0}^{i,-1}$* *is bounded, and there is a positive real constant* $\bar{a}$*, $\bar{b}$, $\bar{q}$, and $\bar{r}$* *that satisfy $\alpha_{t-1}^{i}F_{t-1}^{i}{\left(\alpha_{t-1}^{i}F_{t-1}^{i}\right)}^{T}\leq \bar{a}I_{n}$* *and $\bar{q}\leq Q_{t-1}^{i}$* *, we can obtain*

(68) $$\zeta_{t}^{i,-1}\leq \zeta_{t|t-1}^{i,-1}\leq p_{k}^{-1}I_{n}$$

*where $p_{t}^{-1}=\lambda_{\max}(\zeta_{0}^{i}){\bar{a}}^{t}+\bar{q}\sum_{k=0}^{t-1}{\bar{a}}^{k}$, and $p_{0}^{-1}=\lambda_{\max}(\zeta_{0}^{-1})$, where $\lambda_{\max}(\zeta_{0}^{-1})$ is the maximum eigenvalue of $\zeta_{0}^{-1}$.*

Moreover, $p_{t}^{-1}<p=\frac{\max[p_{0}^{-1},\bar{q}]}{1-\bar{a}}$ when $\bar{a}<1$.

**Proof of Theorem 4.**  From Equation (57) and Lemma 2, we can obtain

(69) $$\zeta_{t}^{i}\geq \zeta_{t|t-1}^{i}\geq \zeta_{t-1}^{i}\geq \cdots \geq \zeta_{0}^{i}$$

So, we only need to prove that $\zeta_{0}^{i,-1}=P_{0}^{i}$ is bounded. We use mathematical induction to prove this conclusion.

For t=0, from Equation (69), we can obtain $\zeta_{0}^{i,-1}\leq \lambda_{\max}(\zeta_{0}^{i,-1})I_{n}=p_{0}I_{n}$. Assume $\zeta_{t-1}^{i,-1}\leq p_{k-1}I_{n}$ at t−1. Next, we only need to prove that the above conclusion is still true at time t.

From Equations (55) and (57), we can obtain

(70) $$\begin{array}{ll} \zeta_{t}^{i} & \geq {\left[\alpha_{t-1}^{i}F_{t-1}^{i}{\left(\zeta_{t-1}^{i}\right)}^{-1}{\left(\alpha_{t-1}^{i}F_{t-1}^{i}\right)}^{T}+Q_{t-1}^{i}\right]}^{-1}\\ & \geq {\left[p_{t-1}^{-1}\alpha_{t-1}^{i}F_{t-1}^{i}{\left(\alpha_{t-1}^{i}F_{t-1}^{i}\right)}^{T}+Q_{t-1}^{i}\right]}^{-1}\\ & \geq {\left(p_{t-1}^{-1}\bar{a}+\bar{q}\right)}^{-1}I_{n}\\ & \geq {\left[\lambda_{\max}(\zeta_{0}^{i}){\bar{a}}^{t}+\bar{q}\sum_{k=0}^{t-2}{\bar{a}}^{k+1}+\bar{q}\right]}^{-1}I_{n}=p_{t}^{-1}I_{n} \end{array}$$

When $\bar{a}<1$, we have

(71) $$p_{t}^{-1}=\lambda_{\max}(\zeta_{0}^{-1}){\bar{a}}^{t}+\bar{q}\sum_{k=0}^{t-1}{\bar{a}}^{k}$$

(72) $$p_{t}^{-1}\leq \max[\lambda_{\max}(\zeta_{0}^{-1}),\bar{q}]\sum_{k=0}^{t}{\bar{a}}^{k}<\frac{\max[\lambda_{\max}(\zeta_{0}^{-1}),\bar{q}]}{1-\bar{a}}$$

In conclusion, the Proof of Theorem 4 is completed. □

## 5. Simulation

A simulation experiment is designed with a cluster of seven USVs to validate the effectiveness of the algorithm proposed in this paper. The state equation can be modeled as follows:

(73) $$x_{k}=\left(\begin{array}{ccccc} 1 & \frac{\sin\Omega T}{\Omega } & 0 & -\left(\frac{1-\cos\Omega T}{\Omega }\right) & 0\\ 0 & \cos\Omega T & 0 & -\sin\Omega T & 0\\ 0 & \frac{1-\cos\Omega T}{\Omega } & 1 & \frac{\sin\Omega T}{\Omega } & 0\\ 0 & \sin\Omega T & 0 & \cos\Omega T & 0\\ 0 & 0 & 0 & 0 & 1 \end{array}\right)x_{k-1}+w_{k}$$

The measurement equation of the system can be represented by Equation (5), and the communication network topology of the USV cluster is shown in Figure 2.

The initial state $x_{0}^{i}$ and error covariance $P_{0}^{i}$, i∈1~7 of USVs are set as $x_{0}^{1}={\left(0,2,0,4,0.10\right)}^{T}$, $x_{0}^{2}={\left(15,2,30,4,0.10\right)}^{T}$, $x_{0}^{3}={\left(15,2,-30,4,0.10\right)}^{T}$, $x_{0}^{4}={\left(30,2,0,4,0.10\right)}^{T}$, $x_{0}^{5}={\left(45,2,0,4,0.10\right)}^{T}$, $x_{0}^{6}={\left(60,2,30,4,0.10\right)}^{T}$, $x_{0}^{7}={\left(60,2,-30,4,0.10\right)}^{T}$, $P_{0}^{1}=diag(2,0.3,1,0.2,10^{-4})$, $P_{0}^{2}=diag(0,0.2,1,0.1,10^{-4})$, $P_{0}^{3}=diag(1,0.4,0,0.2,10^{-4})$, $P_{0}^{4}=diag(1,0.1,0,0.3,10^{-4})$, $P_{0}^{5}=diag(1.2,0.25,1,0.3,10^{-4})$, $P_{0}^{6}=diag(1,0.1,1.5,0.3,10^{-4})$, and $P_{0}^{7}=diag(1,0.2,2,0.1,10^{-4})$.

The system noise settings for the seven USVs are set as $w_{t}^{i},\;i\in 1\sim 7$, which are Gaussian white noise with a mean of zero, and the covariances are set as $Q_{t}^{1}=diag(2,1,2,3,10^{-6})$, $Q_{t}^{2}=diag(1.5,4,1.5,4,10^{-6})$, $Q_{t}^{3}=diag(2.5,3,2.5,3,10^{-6})$, $Q_{t}^{4}=diag(1,2,1,2,10^{-6})$, $Q_{t}^{5}=diag(2,3.5,2,3,10^{-6})$, $Q_{t}^{6}=diag(1.8,2,1.8,3,10^{-6})$, and $Q_{t}^{7}=diag(2.2,1,2.2,3,10^{-6})$.

The measurement noise settings for the seven USVs are set as $v_{t}^{i},\;i\in 1\sim 7$, which are Gaussian white noise with a mean of zero. The position measurement noise covariances are the same as the $Q_{t}^{i}$ setting, the distance measurement noise covariances are set as 0.5, and the relative angle measurement noise covariances are set as 1°.

The probabilities of each USV measurement delay are set as $p_{t}^{1}=0.85$, $p_{t}^{2}=0.9$, $p_{t}^{3}=0.83$, $p_{t}^{4}=0.8$, $p_{t}^{5}=0.78$, $p_{t}^{6}=0.93$, and $p_{t}^{7}=0.88$.

The root mean-squared error (RMSE) is defined as

(74) $$RMSE(t)=\sqrt{\frac{1}{N}\sum_{n=1}^{N}{\left(x_{t}^{i}-{\hat{x}}_{t}^{i}\right)}^{2}}$$

where ${\hat{x}}_{t}^{i}$ is the USV i state at time t, and N=50 denotes the number of iterations of the Monte Carlo simulation.

**Figure 3:**
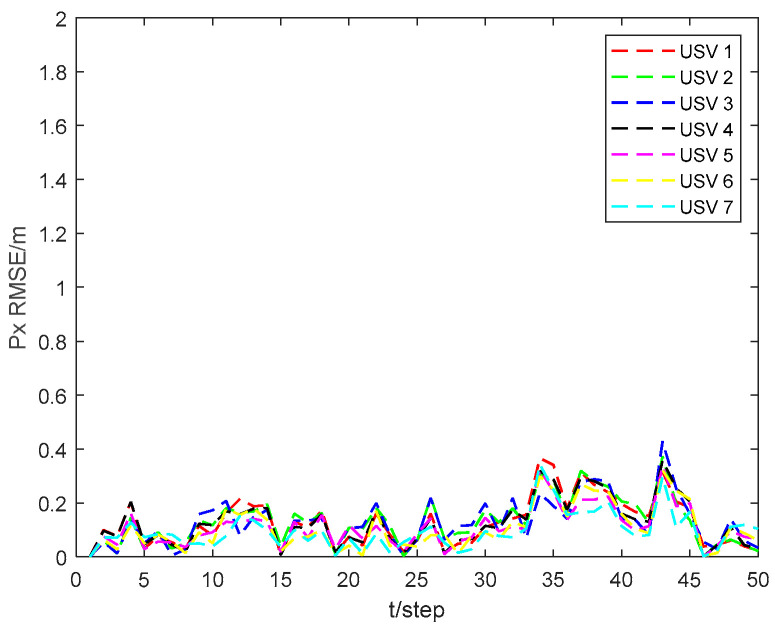
Position state $p_{x}^{i}$ RMSEs of CKHCF for USVs 1–7.

**Figure 4:**
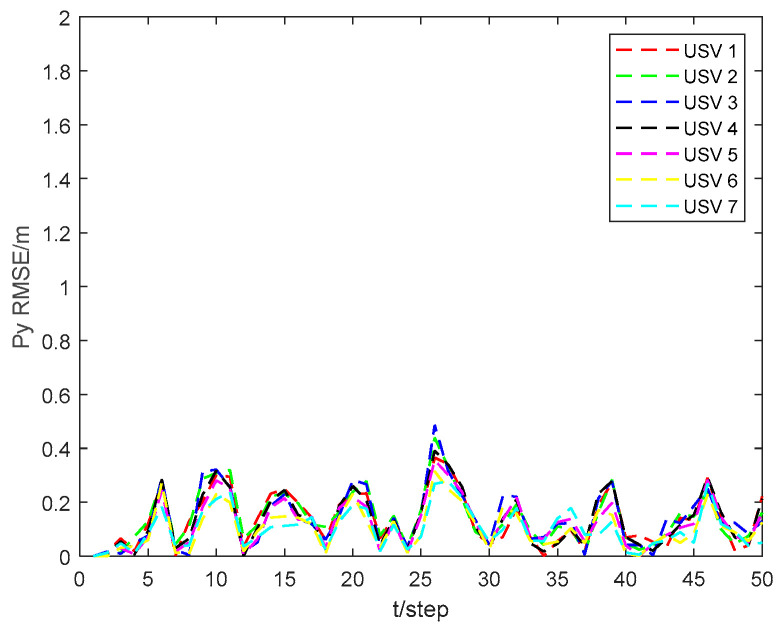
Position state $p_{y}^{i}$ RMSEs of CKHCF for USVs 1–7.

Figure 3 and Figure 4 show the position state $p_{x}^{i}$ and $p_{y}^{i}$ RMSEs of the CKHCF for USVs 1–7. As can be seen from the figures, USVs with diverse initial parameters can maintain localization consistency through information exchange and fusion within the cluster.

**Figure 5:**
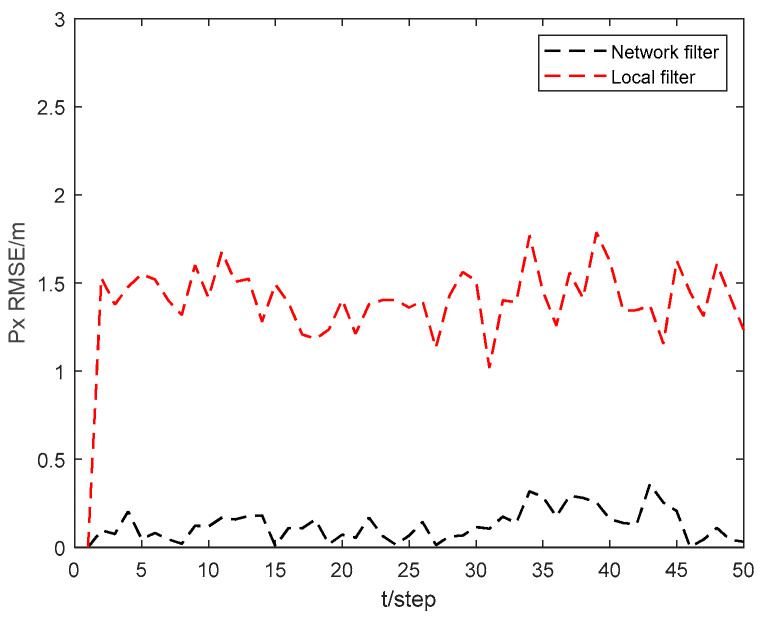
Position state $p_{x}^{4}$ RMSEs of local filter and network filter for USV 4.

**Figure 6:**
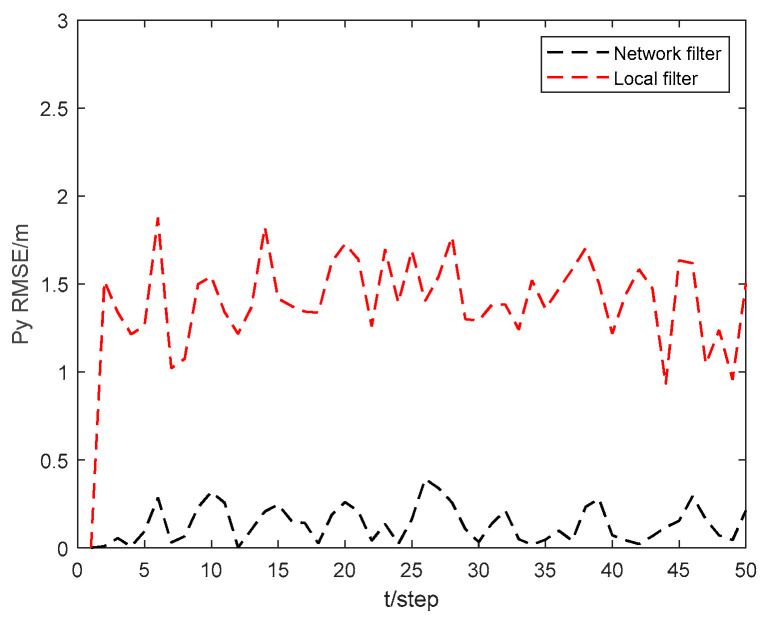
Position state $p_{y}^{4}$ RMSEs of local filter and network filter for USV 4.

Figure 5 and Figure 6 show the position state $p_{x}^{4}$ and $p_{y}^{4}$ RMSEs of the local filter and network filter for USV 4. As can be seen from the figures, the state RMSEs obtained by USV 4 through collaborative localization in the USV cluster network can be significantly reduced compared to those obtained by USV 4 through local filtering. This demonstrates that the collaborative localization network of the USV cluster can greatly improve the localization accuracy of the USVs within the cluster.

**Figure 7:**
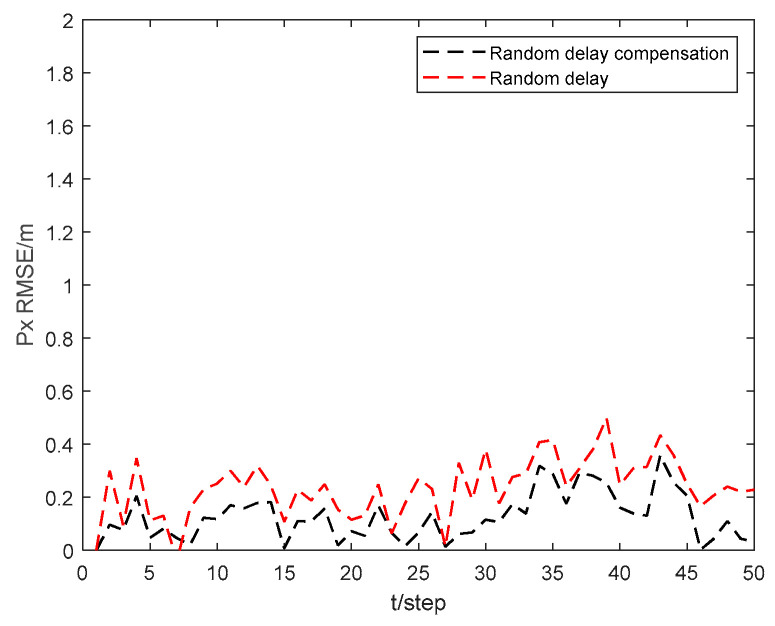
Position state $p_{x}^{4}$ RMSEs of random delay compensation and without compensation for USV 4.

**Figure 8:**
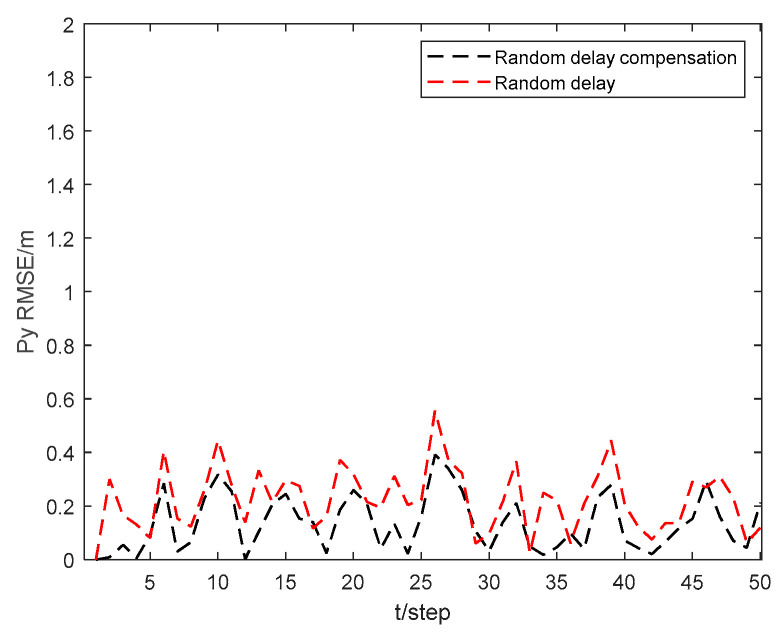
Position state $p_{y}^{4}$ RMSEs of CKHCF with random delay compensation and without compensation for USV 4.

Figure 7 and Figure 8 show the position state $p_{x}^{4}$ and $p_{y}^{4}$ RMSEs of the CKHCF with random delay compensation, as well as without compensation for USV 4. As can be seen from the figures, random delay can effectively enhance the localization accuracy of the USV within the cluster.

**Figure 9:**
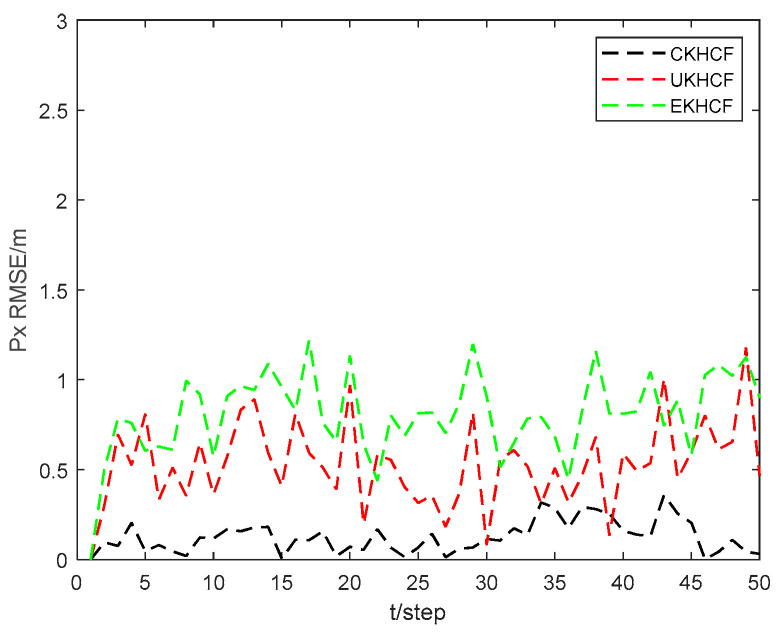
Position state $p_{x}^{4}$ RMSEs of CKHCF, UKHCF, and EKHCF for USV 4.

**Figure 10:**
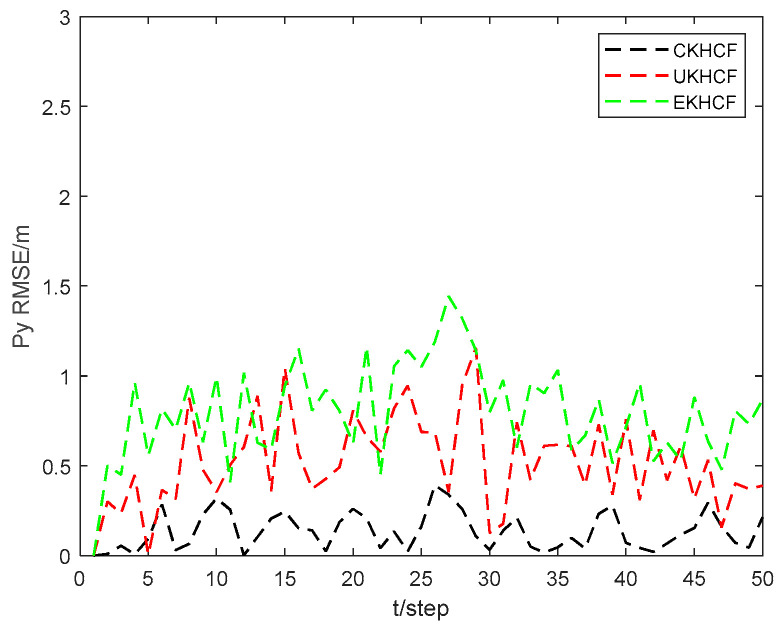
Position state $p_{y}^{4}$ RMSEs of CKHCF, UKHCF, and EKHCF for USV 4.

Figure 9 and Figure 10 show the position state $p_{x}^{4}$ and $p_{y}^{4}$ RMSEs of the CKHCF, unscented Kalman hybrid consensus filter (UKHCF), and EKHCF for USV 4. As can be seen from the figures, the EKHCF has relatively higher state RMSEs, UKHCF has relatively lower state RMSEs, and CKHCF has the lowest state RMSEs. This is because the EKHCF neglects higher-order terms during linear approximation, leading to reduced filtering accuracy. On the other hand, the UKHCF and CKHCF utilize an approximate probability density function method, resulting in higher filtering accuracy. However, the UKF loses some statistical properties of Sigma points related to the posterior distribution of nonlinear functions, thereby reducing system estimation accuracy.

**Figure 11:**
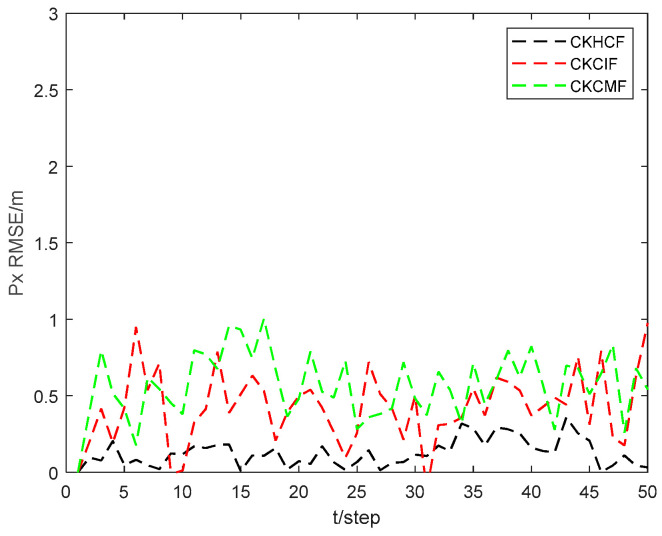
Position state $p_{x}^{4}$ RMSEs of CKHCF, CKCIF, and CKCMF for USV 4.

**Figure 12:**
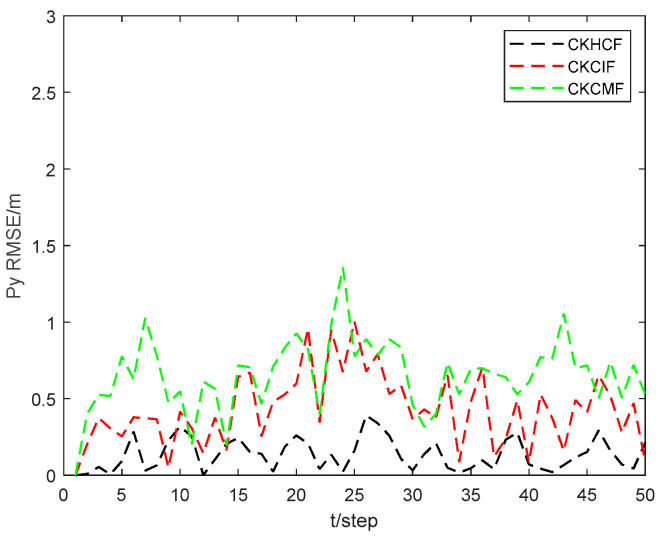
Position state $p_{y}^{4}$ RMSEs of CKHCF, CKCIF, and CKCMF for USV 4.

Figure 11 and Figure 12 show the position state $p_{x}^{4}$ and $p_{y}^{4}$ RMSEs of the CKHCF, cubature Kalman consensus information filter (CKCIF), and cubature Kalman consensus measurement filter (CKCMF) for USV 4. As can be seen from the figures, the CKHCF algorithm exhibits the best estimation accuracy. This is because the CKCIF algorithm ignores the correlation between local estimations, while the CKCMF algorithm is unstable when there are fewer consensus steps. The CKHCF algorithm integrates the estimation and contribution of information from each USV separately, updating the information pairs using the fused results. This approach not only combines the advantages of the CKCIF and CKCMF but also overcomes their shortcomings.

## 6. Conclusions

In this paper, we design a collaborative localization algorithm CKHCF that is suitable for USV cluster systems with random measurement delays. Each USV exchanges two sets of information with all its neighbors, and after calculating the received information, the fusion of all information is used to obtain positioning based on global measurements. The proposed algorithm was proven to maintain consistency and boundedness at all times and in every consensus step through mathematical induction. In future research, we will focus on investigating how to utilize existing CKF algorithms [32,33,34,35,36] to mitigate measurement interference caused by factors such as wind, waves, and ocean currents.

## Data Availability

Data are available on request due to privacy or ethical restrictions. The data presented in this study are available on request from the corresponding author.
